# Medical and Paramedical Students’ Positive Experiences With Interprofessional Simulation: A Questionnaire Study

**DOI:** 10.7759/cureus.71136

**Published:** 2024-10-09

**Authors:** Alina Lehto, Paula Heikkilä, Anna Sepponen-Lavikko, Jari Laurikka, Tarja Vanhatalo-Suonurmi, Lasse Tervajärvi, Nina Hutri

**Affiliations:** 1 Paramedic Nursing, Tampere University of Applied Sciences, Tampere, FIN; 2 Faculty of Medicine and Health Technology, Tampere University, Tampere, FIN; 3 Anesthesia, Tampere University Hospital, Tampere, FIN; 4 Pediatric Nursing, Tampere University of Applied Sciences, Tampere, FIN

**Keywords:** education, interprofessional simulation, learning experiences, medical students, paramedical students, teamwork

## Abstract

Introduction: Simulation is a common and beneficial learning method in healthcare education. Interprofessional simulation combines both interprofessional collaboration and simulation, and it has been found to improve teamwork, interprofessional collaboration, and patient safety. This study aimed to evaluate medical and paramedical students’ attitudes toward interprofessional simulation and their competence to act in acute situations.

Methods: High-fidelity interprofessional acute simulations focused on internal medicine emergencies and both child and adult resuscitation are part of medical and paramedical curricula at Tampere University and Tampere University of Applied Sciences. A total of 120 final-year medical students and 34 third-year paramedical students participated in these simulations in spring 2021 and were asked to answer pre- and post-questionnaires. The response rates were 91% (n=140) for the pre-questionnaire and 86% (n=132) for the post-questionnaire. A mixed method was used for analysis.

Results: The students’ attitudes toward interprofessional simulations were positive, and interprofessional simulation was considered an effective and safe learning method. Students experienced interprofessional simulations as an excellent learning method. Before the interprofessional simulations, 66% of the students rated their skills to act in acute situations as good or very good, and after the interprofessional simulations, 95% of the students rated their skills to act in acute situations as good or very good. Effective debriefing and positive feedback were considered important for learning. Interprofessional simulations promoted understanding of other professions and interprofessional communication. When the students self-evaluated their skills to act in acute situations, improvement was detected.

Conclusions: Medical and paramedical students appreciated interprofessional collaboration. The students considered interprofessional simulation as an effective learning method and promoting the understanding of other professions. Results confirm that interprofessional simulation is also a good way to teach non-technical skills such as communication and teamwork.

## Introduction

Simulation is a commonly used learning method in healthcare education. It can be determined as real-life imitating learning situations, teaching different skills and procedures in a safe environment without harming real patients [1,2]. Repeated exposure to simulation learning can increase students’ confidence and active participation and improve students’ open self-expression and reflection during debriefing [3-5]. Debriefing in the context of simulation can be defined as a facilitator-guided conversation where the learner is at the center, reflecting on their own actions and decisions, receiving feedback, and learning from the simulation experience [6,7].

Simulation is based on Kolb’s concept of experimental learning, which is an important theoretical foundation of simulation [8]. Kolb’s [9] experiential learning cycle consists of four stages of learning: concrete experience, reflective observation, abstract conceptualization, and active experimentation, all stages being necessary for the learning process to happen. According to Kolb, the learner’s reflections are grounded in concrete experiences. Formed reflections are assimilated and structured into abstract concepts that form new implications. The implications are the basis for active experimentation, where they are tested and, if proven useful, used in new experiences [9]. So students apply the knowledge and skills they have learned earlier to the simulations.

Interprofessional simulation combines simulation and interprofessional collaboration, a team of students or professionals from different healthcare fields working together toward a common goal [10-12]. Interprofessional simulation improves teamwork, leadership, understanding of different roles, and how to work effectively in interprofessional teams [1,4,13,14]. Teamwork and collaboration are crucial when it comes to safe patient care [4,12]. Our study aimed to evaluate medical and paramedical students’ experiences and attitudes toward interprofessional simulations and their ability to act together in emergency situations.

## Materials and methods

The present survey study was carried out at the Tampere Centre for Skills Training and Simulation as a collaboration between Tampere University and Tampere University of Applied Sciences. The Tampere Centre for Skills Training and Simulation is a training center owned by Tampere University, Tampere University of Applied Sciences, and Pirkanmaa Hospital District, providing simulation spaces and rooms for learning where students and professionals in medicine and other healthcare-related fields can work on their practical skills. The study was approved by Tampere University and Tampere University of Applied Sciences (approval number: 21/TAY/TAMK). Participation in the interprofessional simulations was compulsory for medical and paramedical students while responding to the questionnaires was voluntary. All responses were gathered anonymously to maintain the confidentiality of the participants.

Final-year medical students (n=120) and third-year (studies take four years to complete) paramedical students (n=34) participated in high-fidelity interprofessional simulations focused on internal medicine emergencies and resuscitation of a child and adult. Both internal medicine and pediatric simulations included two simulation cases, and the duration was 2.5 hours per session. In the pediatric simulations, there was a skills training session (airway management, compressions during child resuscitation, and managing intraosseous access) before the simulation cases. The learning objectives of the interprofessional simulations were the ABCDE protocol, teamwork, and interprofessionalism. There were 10-12 medical and two to three paramedical students per group. The interprofessional simulations were planned and executed by the teachers of medicine and nursing and teaching coordinators working at the Tampere Centre for Skills Training and Simulation.

The questionnaires measuring the students’ experience and attitudes toward interprofessional simulations and skills to act in emergency situations were sent to the students before and after the interprofessional simulations. The questionnaires featured 7-point Likert scale (1 = very poor, 2-3 = poor, 4 = average, 5-6 = good, 7 = very good) questions and open-ended questions.

The mixed method was used for analyses. Data consisting of open-ended answers was analyzed with inductive, qualitative content analysis. The inductive approach is based on finding similarities and differences in the data, describing the researched phenomenon, and moving from the specific to the general [15]. The answers have been analyzed and classified according to the qualitative method. During the analysis, the material is condensed into themes, from which the research results emerge. Otherwise, the quantitative approach was used to analyze the numerical data. The descriptive analysis was carried out using SPSS Statistics version 26 (IBM Corp. Released 2019. IBM SPSS Statistics for Windows, Version 26.0. Armonk, NY: IBM Corp.), and the data was presented with numbers and percentiles or standard error (SE) or with mean and standard deviation (SD) if the variable was continuous.

## Results

The response rate of the study was 91 % (n=140) in the pre-questionnaire and 86 % (n=132) in the post-questionnaire. In the pre-questionnaire, four-fifths of the respondents were medical students (n=113), and one-fifth were paramedical students (n=27). Respondents’ mean age was 27 years (SD 3). The content analysis classified the data into three classes: attitudes, experiences, and interprofessional learning.

Attitudes

Based on the pre-questionnaire’s answers, simulation was an effective learning method. Simulations help to recognize own strengths and weaknesses. Interprofessionalism helps the learning process because the learning situation is more realistic and there is a possibility to learn from other professionals. Simulation was considered exciting but also a safe situation where it’s allowed to make mistakes. Few students were concerned about other students’ reactions and found simulations exciting. Few students regarded simulation as unrealistic and like being on a test. Those attitudes did not appear in the post-questionnaire. Positive feedback is essential and improves the sense of competence. Previous experiences with simulations affected the attitudes toward following simulations.

Simulation learning is one of the best learning methods in my opinion. Debriefing has usually been rewarding.

Experiences

Simulation was experienced as an effective way to learn. It improved confidence, firmed already existing knowledge and skills, and strengthened the engram of what’s been learned. The students had very few previous experiences with simulation, and so it was experienced that the curricula included too little simulation learning. Some of the students regarded that those previous simulations had been placed at a too early phase of studies. Interprofessional simulations caused uncomfortable emotions beforehand for some students, but after the simulations, the benefits were considered remarkably higher. Group dynamics and interaction with each other were considered important factors in the experience of success. The group size was also important, as the students felt that too big groups disturbed learning. Debriefing was an important part of the simulation process and improving students’ confidence. Few students hoped for a longer time for debriefing.

Interprofessional learning

A desire for more interprofessional education and simulation existed, as initially, students had only a few previous experiences with interprofessional learning. The timing for simulation (the final stage of studies) was considered advisable since focusing too much on technical skills wasn’t regarded as beneficial. Students (both medical and paramedical) reported that the interprofessional simulations increased their trust and positive attitudes toward other student groups. Interprofessionalism made simulations more realistic and allowed learning from other students. Some medical students acted as nurses during simulations. Few of them experienced that it’s important to be in their own profession’s role. In some debriefing sessions, there were no nursing/paramedical teachers, which may explain why few paramedical students regarded the interprofessional simulations as too doctor-focused and debriefing insufficient in the context of nursing.

Interprofessional learning is a great idea. The group sizes should be small enough and everyone should be able to participate and act in their own, real-life professional role. I think that you can learn about other professions’ assignments even if you act in your own role, although it’s nice to be in another occupational group for a moment.

Self-evaluation

**Figure 1:**
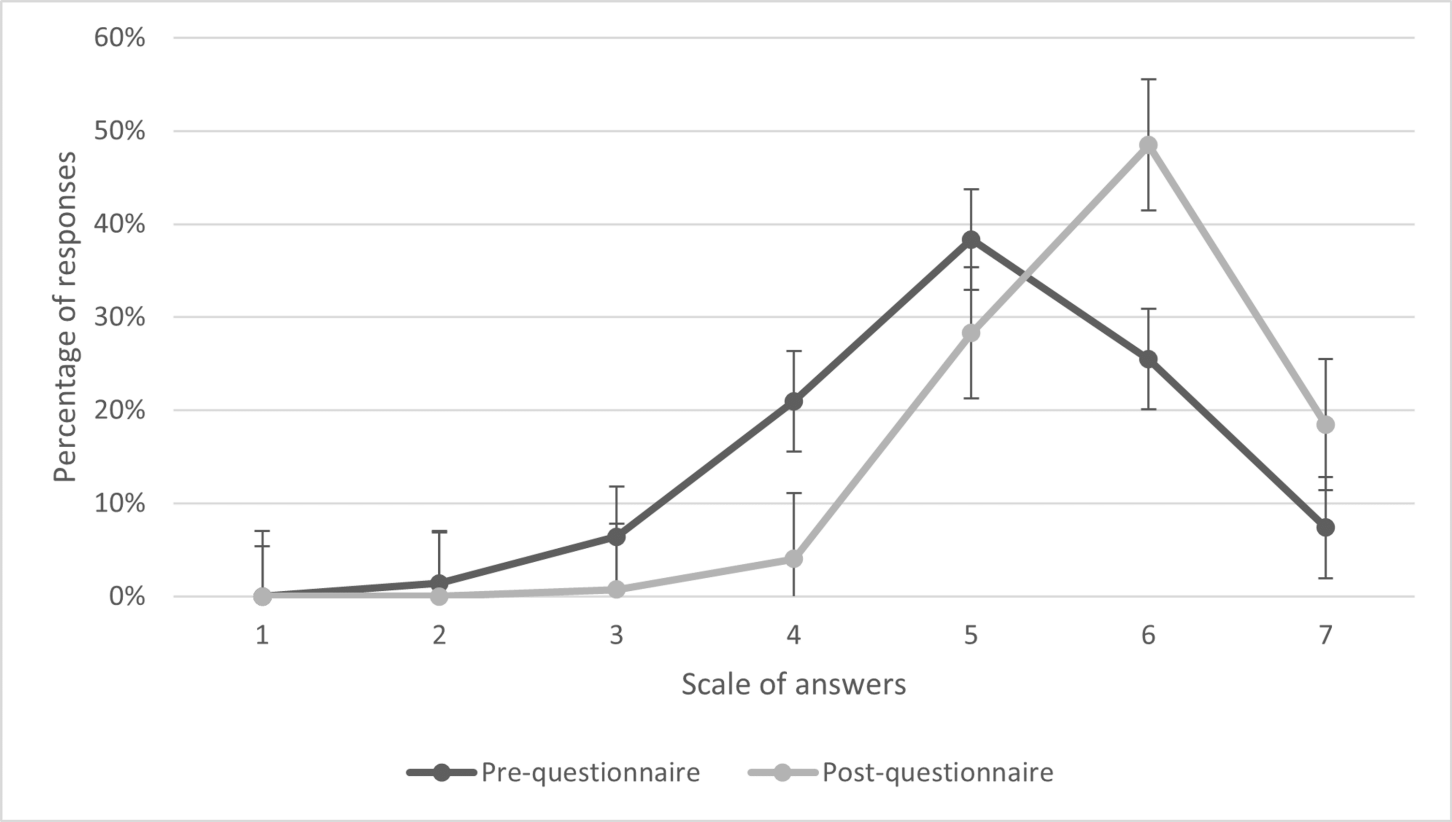
The students’ self-assessment of their competence to act in acute situations before and after the interprofessional simulations. Answers on a scale from 1 to 7 represent “very poor” (=1), “poor” (=2-3), “average” (=4), “good” (=5-6), and “very good” (7). The graph represents the percent of responses, and error bars represent standard errors (SE)

Students considered their competence to act in acute situations better after the interprofessional simulations (Figure 1). Before the interprofessional simulations, 66% (n=92) of the students rated their skills to act in acute situations as good or very good, 22 % (n=31) as average, and 12% (n=17) as poor or very poor. The mean rating before the interprofessional simulations was 4.9. After the interprofessional simulations, 95% (n=125) of the students rated their skills to act in acute situations as good or very good, 4 % (n=5) as average, and less than one percent (n=2) as poor. The mean rating after the interprofessional simulations was 5.8. As seen in Figure 1, there was a significant increase in students’ self-assessment of their competence.

## Discussion

Adequate cooperation, communication, and skill sharing are crucial for high-quality patient-safe healthcare (International Nursing Association for Clinical Simulation and Learning [16]). Interprofessional simulation enhances those non-technical skills, provides a chance to practice acute situations [13,14,17], and makes undergraduate students more prepared for working in a real-life interprofessional team [12,18].

The current study evaluated medical and paramedical students’ attitudes and experiences in interprofessional simulation and their ability to act in acute situations. Medical and paramedical students’ attitudes toward interprofessional simulation were positive. Simulation was considered an effective learning method. The students experienced the interprofessional simulations effectively, as simulation is suitable for recognizing one’s own strengths and weaknesses and provides high-quality learning experiences. Debriefing was stated as an essential part of the learning process. Interprofessionalism was important to the students. It enhanced confidence in different professions and improved the understanding of other professions’ expertise and role in an interprofessional team. After the interprofessional simulations, the students considered their competence to act in acute situations better.

The students exhibited favorable attitudes toward interprofessionalism and the practice of interprofessional simulation. The students appreciated the possibility of training in interprofessional collaboration and, therefore, considered interprofessional simulations important and thought that more interprofessional education was needed. This is in line with previous findings; students appreciate simulation, teamwork, and interprofessional collaboration [18-20]. Labrague et al. [21] found that students’ attitudes toward interprofessional learning improved after simulation. Previous experiences of simulation affected the students’ attitudes toward simulation. The safe learning situation and positive feedback from teachers were considered very important. Few students were concerned about other students’ reactions and found simulations exiting beforehand, and a few students also regarded simulation as unrealistic and like being on a test, but those attitudes did not appear in the post-questionnaire. This may indicate that the interprofessional simulations have been successfully organized.

Students viewed interprofessional simulations as effective and secure learning environments that not only boosted their confidence but also reinforced existing knowledge and skills, enhancing the retention and application of learned concepts. The interprofessional simulations caused excitement before the interprofessional simulations for some of the students, but the benefits were considered remarkably higher after. Jakobsen et al. [22] similarly found that students experienced strong emotional arousal during simulation. According to Li et al. [23] and Toyota [24], emotionally distinctive activity can affect recollection (slowly retrieving details associated with previously presented matter) more positively than emotionally neutral activity, as emotions modulate brain function during recollection and emotional enhancement of memory is dependent on emotional activity. This indicates that simulation can be effective but also stressful to participants.

The students experienced that interprofessional simulations promote interprofessionalism and help to understand other professions’ education and skills. This is consistent with previous research: interprofessional simulation improves communication and a better understanding of both their own and the other profession’s expertise and role in an interprofessional team [1,13,22,25]. The professional needs of each occupation group have to be taken into consideration, and this creates an additional need for teaching resources. WHO [12] stated that interprofessional education gives undergraduate healthcare professionals the readiness to work in a collaborative team, so the need for more interprofessional education and, therefore, more resources for interprofessional education should be addressed.

Debriefing is commonly experienced as important by students [4,6,22]. Similarly, the students of this study considered debriefing an important part of the learning process and increased their confidence. Koo et al. [26] and Reed et al. [27] have previously emphasized that it is important that experts from every speciality are involved in interprofessional simulations. The students from different professions have different professional needs and, hence, observe the details of the simulation scenarios from different perspectives. Unfortunately, a nursing/paramedical teacher couldn’t participate in all debriefing sessions. Therefore, few paramedical students considered debriefing insufficient.

The students gained confidence in their own skills, and their competence to act in acute situations improved. Before the interprofessional simulations, the mean rating was 4.9, while the mean rating after was 5.8. After the interprofessional simulations, less than one percent of the students rated their skills to act in acute situations as poor. The rest rated their skills to act in acute situations as average, good, or very good. This could indicate that the students considered that they have quite moderate skills to act in acute situations. Similarly, the students reported that their technical skills improved. The students’ increasing confidence levels in general and with patient care skills after simulation activities have been detected earlier [3,28]. However, it has been argued that increased self-confidence can be transformed into increased competence [28,29]. Liaw et al. [30] carried out an intervention study evaluating if students’ self-reported confidence and knowledge levels indicate clinical performance. Both the intervention group and the control group reported higher confidence levels after simulation, although the intervention group scored significantly higher in knowledge and clinical performance.

In our study, the response rate was surprisingly high. Therefore, we can say that the data is quite reliable. The number of students participating in this study was remarkably high considering that a qualitative approach was used. There was enough data to additionally use a quantitative method. The limitation of this study is that the results are grounded on students’ self-expression and self-assessment, providing only subjective data on the benefits of interprofessional simulation, so it did not objectively measure students’ attitudes toward interprofessional simulation and their competence to act in acute situations. However, studies with similar research designs commonly use self-evaluation; subjective data from students is necessary for evaluating genuine experiences and, therefore, the benefits of interprofessional simulation. Even though the simulations are challenging to implement, they prepare students for working life.

## Conclusions

The medical and paramedical students appreciate interprofessional collaboration and consider interprofessional simulation effective. Interprofessional simulation improved collaboration and confidence in other professions. This study consolidates the impression that interprofessional education is a good learning tool for non-technical skills like communication and teamwork. Critical elements for successful interprofessional simulations include sufficient time for debriefing, opportunities to perform in one's professional capacity, and ensuring appropriate group sizes. The study confirms that although interprofessional simulation requires adequate planning and resources, the use of interprofessional simulations should be encouraged.

## Appendices

Pre- and post-questionnaire

1. Gender

a. Female

b. Male

2. Age

3. I’m a

a. Medical student

b. Paramedical student

4. I’m interested in the following specialization options/jobs (mention max. 3)

5. I have previous education…

a. From the health sector

b. From another sector

c. I don’t have previous education

6. A 5-year-old child has gone lifeless in the emergency room. You start CPR. What is the most common cause of lifelessness in children?

a. Respiratory failure due to various causes

b. Arrhythmia due to various causes

c. Heart failure due to various causes

7. How is it recommended that children be given medication during resuscitation?

a. Only into a vein

b. Only intraosseally

c. For intubation tube only

d. Intravenous or intubation tube

e. Intravenously or intraosseally

f. Intravenously, intraosseally, or intubation tube

8. Medical resuscitation: improving the prognosis of a lifeless patient:

a. Early securing of the airway immediately prior to rhythm detection

b. Early defibrillation when the initial rhythm is PEA

c. Immediate and high-quality PPE and early defibrillation

d. Immediate opening of the vascular connection for the administration of adrenaline

e. Early administration of sodium bicarbonate routinely for metabolic acidosis

9. A 56-year-old man has hypertension and now a sudden severe pain between the shoulder blades, which causes him to pass out. The heart rate is fast, steady at 105/min, the man is now conscious but very painful. You find that the pulse on the right wrist is weak compared to the left. You suspect a circulatory disorder. What is the most likely diagnosis?

a. Arterial embolism of the right hand only

b. Aortic dissection and reduced blood flow to the right arm

c. Stroke in the left hemisphere

d. Atrial fibrillation

10. A first-time birth comes to the emergency room H36+5. She's had cramp-like stomach pains for a few hours, but since the due date is still weeks away, she didn't think it could be labor contractions. In the internal examination, you find that the cervix is ​​completely open and the head presents itself at the height of the mid-pelvis, i.e. at about the level of the spine. The maternity hospital is half an hour away. How do you work?

a. You give medicine that the birth stops and you send the ambulance to the maternity hospital

b. You send the ambulance to the maternity hospital but make sure that the ambulance staff can handle childbirth if necessary

c. You state that the birth will take place so soon that it is not possible to send for an ambulance, and you begin to actively push the mother

11. The ambulance transporting the rebirther to the hospital stops at the one along the way to the health center, because the laborer is starting to strain. The pushing phase is quick and problem-free, and with almost no push-out, a toned baby girl is born, who will soon begin to growl. But what do you do with the umbilical cord? Choose the best option from the following.

a. Nothing, the baby is still getting oxygenated blood through the placenta

b. You immediately close the umbilical cord with one pair of pliers 2 cm from the baby's navel and cut the umbilical cord with scissors from the side of the placenta

c. When the umbilical cord stops pulsating, you close the 10 cm end of the umbilical cord with two forceps and cut the umbilical cord with the forceps between

12. I have familiarized myself with the Resuscitation Care recommendation (Käypä Hoito)

a. Yes

b. No

13. I am familiar with the literature on acute cardiology

a. Yes

b. No

14. I am familiar with the course of normal childbirth

a. Yes

b. No

15. I know how to act… (Likert scale, 1-7)

a. In a child resuscitation situation

b. In an adult resuscitation situation

c. In acute cardiology situations

d. In travel birth

16. Free comments/assessment of your own knowledge

17. I can … (Likert scale, 1-7)

a. Act in a child resuscitation situation

b. Control of the child's airways

c. Mask ventilate the child

d. Use alternative airway management tools (ventilation/intubation) in acute cardiology situations

e. Act in an adult resuscitation situation

f. Adult airway management

g. Mask ventilate the adult

h. Use alternates

i. Airway management equipment (breathing/intubation)

j. Act in travel birth

k. Push out a breech baby

l. Take care of the newborn

m. Care for the mother in the third stage of labor

18. I can perform PPE-D (compression-blow resuscitation and defibrillation) (Likert scale, 1-7)

a. I know the child's correct resuscitation rhythm and stick to it

b. I know how to perform CPR for a child

c. I know the importance of defibrillation in children

d. I know the correct CPR rhythm for an adult and I stick to it

e. I know how to perform CPR for an adult

f. I know the importance of defibrillation in adults

19. Free comments/assessment of your own artistic skills

20. Your attitude toward simulation learning and acute care (Likert scale, 1-7)

a. I like emergency work and acute patient situations

b. I dare to act in acute situations

c. The simulation is well suited for learning acute care situations

d. Simulation is an effective way to learn

21. Describe your attitudes toward simulation learning and acute care

22. Your experience with simulation learning and acute care (Likert scale, 1-7)

a. I have benefited from studying simulation

b. My experiences with simulation lessons are positive

c. I have worked in acute care situations

d. My experiences with acute care situations are positive

23. What kind of experiences do you have with simulation learning and acute care?

24. Interprofessional learning (Likert scale, 1-7)

a. I have often participated in interprofessional learning events

b. Interprofessional study is important

c. I have benefited from interprofessional lessons

d. My experiences with interprofessional lessons are positive

25. What kind of experiences do you have with interprofessional learning?
